# An Unbiased Scientific Record Should Be Everyone's Agenda

**DOI:** 10.1371/journal.pmed.1000038

**Published:** 2009-02-24

**Authors:** The PLoS Medicine Editors

## Abstract

This month's editorial discusses ways in which the process of publishing scientific research can be inappropriately influenced by varied forms of bias and the effects of competing interests. The editors propose five ways in which individuals involved in the publication process can mitigate the effects of biased agendas on the published scientific record.

A large and growing literature details the many ways by which research and the subsequent published record can be inappropriately influenced, including publication bias [1], outcome reporting bias [2], financial [3] and non-financial [4] competing interests, sponsors' control of study data and publication [5], and restrictions on access to data and materials [6]. But it can be difficult for an editor, reading a submitted manuscript, to disentangle these many influences and to understand whether the work ultimately represents valid science.

Any journal has stories to tell of attempts to unduly influence the publication process—such as the author who repeatedly appeals a manuscript's rejection, claiming the reviewers are incompetent and demanding evaluation by a specific list of preferred experts, or the biotech company that refuses to publicly deposit the microarray data underlying their findings. Sometimes distortion of the scientific record may be limited in scope, relating to just one paper. But when a single company funds virtually an entire research agenda on a particular topic, there is the potential for wider and far more damaging distortion. In a detailed analysis of documentation released as part of a class-action lawsuit relating to the drug gabapentin (Neurontin), Kay Dickersin has described “…a remarkable assemblage of evidence of reporting biases that amount to outright deception of the biomedical community, and suppression of scientific truth concerning the effectiveness of Neurontin for migraine, bipolar disorders, and pain…” ([7], summarized in [8]). Here we propose five ways in which authors and editors can mitigate the effects of biased agendas on the published scientific record.

## 1. Recognize and Declare Editorial Interests

Journals generally have policies regarding declaration of competing interests by authors. Similarly, editors' political and scientific views, personal relationships, and professional and financial interests can all conceivably interfere with the objectivity of their decisions.

Even financial pressures on a journal can create conditions for possible editorial bias. For example, journals that own the copyright on the articles they publish, giving the journal the right to an exclusive reprint trade, can sell copies of a single drug trial report for hundreds of thousands of dollars. While journals have been reluctant to publicize the exact amount that they make from such reprint sales, a *Wall Street Journal* article is illustrative of the amount of money that changes hands.

The newspaper reported that the *New England Journal of Medicine* sold 929,400 reprints of a single research article [9] reporting the results of a clinical trial of the painkiller rofecoxib (Vioxx) [10]. These reprints were mostly sold to the drug's manufacturer Merck, bringing in more than US$697,000 [9] in revenue for the journal. Editors, says Richard Smith, “know that publishing such studies is highly profitable, and editors are increasingly responsible for the budgets of their journals and for producing a profit for the owners” [11]. This responsibility, he argues, may provide journals with a motivation for preferentially publishing the outcomes of pharmaceutical trials funded by industry.

One of the many advantages of open-access publishing is that this type of editorial bias is minimized. Work that is published in open-access journals, which apply licenses such as the Creative Commons Attribution License used by PLoS [12], can be freely reused. Journals using such licenses therefore have no exclusive reprint trade, and are protected from the inherent bias towards industry-funded pharmaceutical trials that might otherwise operate.

In order to acknowledge the potential for editorial bias, and defend against it, many journals annually update and publish the editors' competing interests (e.g., [13]). At *PLoS Medicine*, editors must recuse themselves from the decision-making process for papers in which they have some potential competing interest—for example, a personal relationship with the authors.

## 2. Be Aware of Interests Beyond the Commercial

So much has been published relating to the damaging nature of commercial competing interests [3] that it is tempting to ignore the influence of non-commercial interests in research. Yet publications can be influenced by the desire to promote an idea, or a research program, rather than a commercial product. At one, very trivial, level, this might include a journal receiving repeated letters from the same individual, each time outlining their pet theory and its relevance to articles the journal has published. A trickier situation to manage might involve the submission of an article presenting research done by an advocacy organization, whose declared political position might be strengthened by the study reported.

Robust journal policies regarding non-commercial competing interests (e.g., [4]) will at least require declaration of any interests that might influence reporting or review, and that would be influenced—negatively or positively—by publication. Such interests might include personal relationships or professional interactions with authors, editors, or reviewers, and strongly held political or religious views that relate to the work under consideration.

In publishing a research article on the unreliability of protocols for execution by lethal injection [14], we declared our own position in an accompanying editorial: “Each of the editors of *PLoS Medicine* opposes the death penalty. It is not our intention to encourage further research to ‘improve’ lethal injection protocols” [15]. It therefore remains for readers to judge for themselves to what extent the underlying interests of editors as well as authors might have affected the published article.

## 3. Consider Whether There Is a Ghost in the Machine

One of the *PLoS Medicine* editors recently handled a manuscript describing the burden of a disease of poverty. The study was designed, and the paper apparently written up, by the manufacturer of a vaccine, but no employees of this company were named in the paper; the authorship byline included only representatives of a health communications company, along with one individual affiliated with the hospitals where the work was done. Had the manufacturer's employees been listed as authors, together with their contributions and competing interests, any role of the company in directing the research project would have been appropriately evident.

In a debate [16] published in this month's issue of *PLoS Medicine,* three viewpoints lay out a variety of approaches for combating ghostwriting (ghostwriting occurs when individuals who have made a substantial contribution to the research project, or to writing of the article, are not named as authors [17]). All contributors to the debate agree on one thing: a transparent declaration of author contributions is an essential requirement. As part of such a transparency policy, editors can therefore ensure that the individuals responsible for essential roles in research (such as designing the project, carrying out analyses, and writing the paper) are actually named, and their roles and competing interests made clear in the publication.

## 4. Where's the Spin? Remember the Protocol

Many journals now have policies requiring, or recommending, the submission of original protocol documents before papers reporting the results of clinical trials are peer-reviewed [18], as well as policies requiring that authors adhere to the CONSORT guidelines for trial reporting [19]. The intention behind such policies is, firstly, to promote transparency, and secondly, to provide a mechanism to detect within-study selective reporting (a phenomenon whereby some findings are omitted from publications and others selected for inclusion or emphasis, dependent on the direction of their results [19]). In practical terms, these policies enable verification of the study's prespecified objectives and analysis plan, and require clear description of any subsequent changes. Although the vast majority of authors have been happy to send us copies of their protocols, we have occasionally met with resistance.

In one situation, we received a report of a randomized, placebo-controlled trial of a marketed intervention, funded by the manufacturer of the intervention being studied. On requesting the study protocol, we received an e-mail from an individual affiliated with the company, but who was not listed as an author of the paper, asking us to sign a confidentiality agreement barring us from making the protocol available to reviewers, and from publishing it as supporting material, as is our policy for clinical trial papers. In this situation, we and the reviewers would have been unable to properly evaluate the submitted report; we declined to consider the paper further, based on the journal's policy regarding reporting standards for clinical trials.

## 5. Anti-Spin: Consider Whether the Data Are Important Even If the Results Aren't Exciting

The Declaration of Helsinki [20] emphasizes that, in rejecting unexciting papers, editors can contribute to a biased research base. The Declaration notes: “Authors, editors and publishers all have ethical obligations with regard to the publication of the results of research…. Negative and inconclusive as well as positive results should be published or otherwise made publicly available” [20]. Editors have an important role to play in encouraging authors to value their results, irrespective of the study's outcome. For example, in an attempt to impress editors with the importance of a study, authors may overemphasize an intriguing post-hoc subgroup analysis, or may avoid stating that a well-conducted trial was inconclusive in its primary outcomes. Editors can help combat this problem by emphasizing to authors that their data are still publishable if overstated conclusions are appropriately toned down. Access to research protocols, or clinical trial registry records, should also help editors to base their decisions on the importance of the underlying research question, and not on the headline potential of actual results. Journals such as our sister journal *PLoS ONE* (http://www.plosone.org/), which aims to publish all methodologically rigorous, ethically sound, and properly reported work, provide one way to ensure that journals do not become part of the problem of publication bias.

Peer-reviewed publication is the final, essential step in any research project, providing legitimization and credit for the work that has been done. It is the responsibility of everyone involved to ensure that the published record is an unbiased, accurate representation of research. We recognize that today there are many, and increasing, pressures on authors and journals to bias this record. If this pressure is not resisted, journals may increasingly become closer to works of fiction telling the stories dictated by various lobbies rather than works of science. We hope that *PLoS Medicine*'s efforts, and those of many other journals, to promote full transparency will ultimately lead to a more rigorous and unbiased knowledge base.
